# Supplementary material for: Kalmusia variispora (Didymosphaeriaceae, Dothideomycetes) Associated with the Grapevine Trunk Disease Complex in Cyprus
Source: Pathogens. 2025 Apr 28;14(5):428. doi: 10.3390/pathogens14050428 (PMC12113838; doi:10.3390/pathogens14050428)
Supplement: Supplementary file 1 [file pathogens-14-00428-s001.zip › Supplementary Figure S1.pdf]

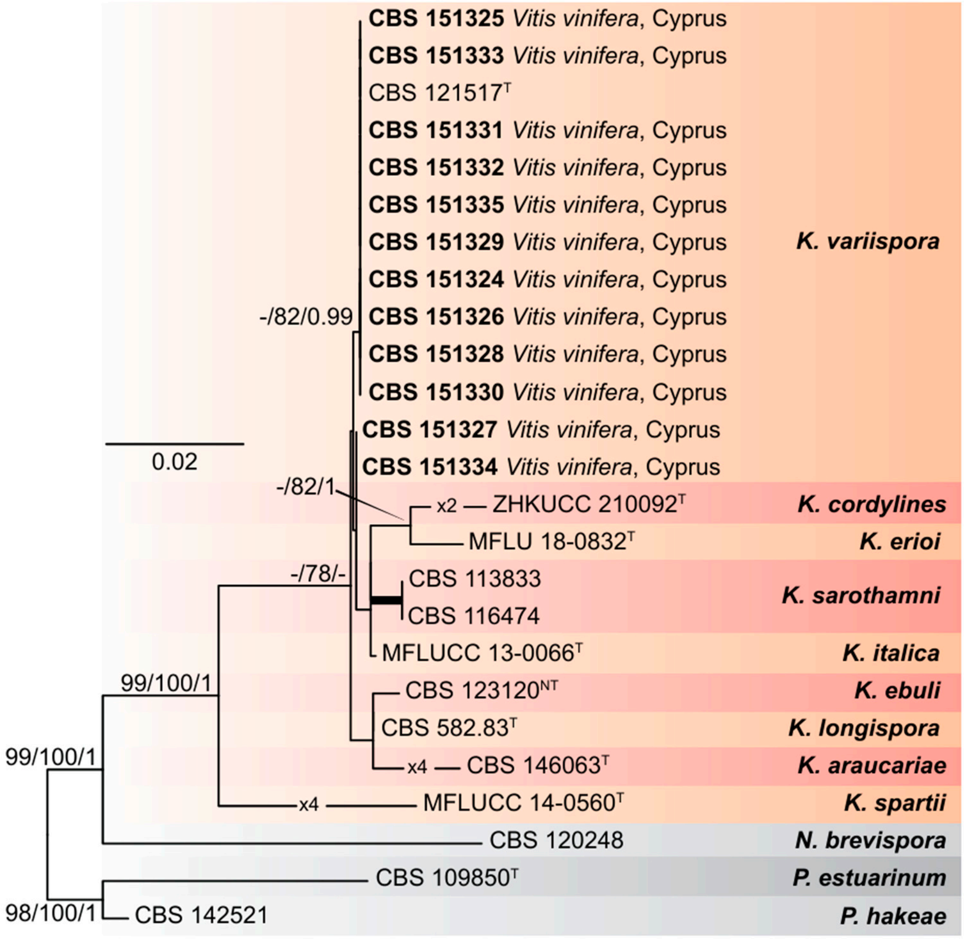

Supplementary Figure S1. Maximum-Likelihood (IQ-TREE-ML) consensus tree inferred from combined rDNA sequences (ITS, LSU, and SSU) of the genus *Kalmusia*. Numbers at the nodes indicate support values (IQ-TREE Uboot, RAxML-BS, and B-PP) above 70% (Uboot and BS) and 0.95 (B). Thickened branches indicate full support (Uboot and BS = 100%, and PP = 1). The scale bar indicates expected changes per site. The tree is rooted to *Neokalmusia brevispora* CBS 120248, *Paraconiothyrium estuarinum* CBS 109850^{T}, and *Paraconiothyrium hakeae* CBS 142521. Ex-neotype and ex-type strains are indicated with ^{NT} and ^{T}, respectively.
