# Supplementary material for: Kalmusia variispora (Didymosphaeriaceae, Dothideomycetes) Associated with the Grapevine Trunk Disease Complex in Cyprus
Source: Pathogens. 2025 Apr 28;14(5):428. doi: 10.3390/pathogens14050428 (PMC12113838; doi:10.3390/pathogens14050428)
Supplement: Supplementary file 1 [file pathogens-14-00428-s001.zip › Supplementary Table S1.pdf]

Supplementary Table S1. *Kalmusia variispora* isolates collected from Cypriot vineyards.

**Codes** [x]	**Province**	**Location of vineyard**	**Cultivar**	**Age (year)**
CBS 151324 (P36)	Paphos	Panagia	Xynisteri	40
CBS 151325 (P95)	Paphos	Axeleia	Superior	25
CBS 151326 (P208)	Paphos	Salamiou	Mavro	40
CBS 151327 (P210)	Paphos	Salamiou	Mavro	40
CBS 151328 (P257)	Paphos	Agios Nikolaos	Mavro	50
CBS 151329 (P192)	Paphos	Agios Ioannis	Mavro	30
CBS 151330 (L63)	Limassol	Kyperounta	Cabernet Sauvignon	20
CBS 151331 (L64)	Limassol	Kyperounta	Cabernet Sauvignon	20
CBS 151332 (L73)	Limassol	Kyperounta	Mavro	50
CBS 151333 (L172)	Limassol	Zoopigi	Xynisteri	40
CBS 151334 (LF7)	Nicosia	Maxairas	Shiraz	8
CBS 151335 (L116)	Nicosia	Polystypos	Mavro	70

[x] Codes of the Cypriot isolates of *Kalmusia variispora* in the culture collection of the Westerdijk Fungal Biodiversity Institute (CBS) and the personal collection numbering in parentheses (Note: The isolates L116, P192, and P210 may also be mentioned in the NCBI database as L116a, P192c, and P210b, respectively).
