# Supplementary material for: Kalmusia variispora (Didymosphaeriaceae, Dothideomycetes) Associated with the Grapevine Trunk Disease Complex in Cyprus
Source: Pathogens. 2025 Apr 28;14(5):428. doi: 10.3390/pathogens14050428 (PMC12113838; doi:10.3390/pathogens14050428)
Supplement: Supplementary file 1 [file pathogens-14-00428-s001.zip › Supplementary Table S2.pdf]

Supplementary Table S2. Primers used in this study.

Primer	Sequence (5′ → 3′)	Direction	Locus	Reference
ITS5	GGA AGT AAA AGT CGT AAC AAG G	Forward	ITS	White et al. 1990
ITS4	TCC TCC GCT TAT TGA TAT GC	Reverse	ITS	White et al. 1990
LR0R	GTA CCC GCT GAA CTT AAG C	Forward	LSU	Rehner & Samuels 1994
LR5	ATC CTG AGG GAA ACT TC	Reverse	LSU	Vilgalys & Hester 1990
NS1	GTA GTC ATA TGC TTG TCT C	Forward	SSU	White et al. 1990
NS4	CTT CCG TCA ATT CCT TTA AG	Reverse	SSU	White et al. 1990
T1	AAC ATG CGT GAG ATT GTA AGT	Forward	*b-tubulin*	O'Donnell & Cigelnik 1997
Bt2b	ACC CTC AGT GTA GTG ACC CTT GGC	Reverse	*b-tubulin*	Glass et al. 1995
5F2	GGG GWG AYC AGA AGA AGG C	Forward	*rpb2*	Sung et al. 2007
7cR	CCC AT(A/G) GCT TG(T/C) TT(A/G) CCC AT	Reverse	*rpb2*	Liu et al. 1999
EF1-728F	CAT CGA GAA GTT CGA GAA GG	Forward	*tef1-a*	Carbone & Kohn 1999
EF2	GGA (G/A)GT ACC AGT (G/C)AT CAT GTT	Reverse	*tef1-a*	O'Donnell & Cigelnik 1998
