# Supplementary material for: Kalmusia variispora (Didymosphaeriaceae, Dothideomycetes) Associated with the Grapevine Trunk Disease Complex in Cyprus
Source: Pathogens. 2025 Apr 28;14(5):428. doi: 10.3390/pathogens14050428 (PMC12113838; doi:10.3390/pathogens14050428)
Supplement: Supplementary file 1 [file pathogens-14-00428-s001.zip › Supplementary Table S3.pdf]

Supplementary Table S3. Characteristics of the different datasets and statistics of phylogenetic analyses used in this study.

Dataset	Partition [x]	Number of sites				Evolutionary models [y]	
		Total	Informative	Invariable	Bayesian unique site patterns	IQ-TREE	Bayesian
**rDNA**	ITS	576	91	376	196	TNe+G4	HKY+G
	LSU	864	15	808	63	TNe+I	GTR+G
	SSU	1021	9	1001	63	K2P+I	HKY+I
**6-loci combined**	*tef1-a*	487	56	343	151	TNe+R3	GTR+I+G
	ITS	573	70	447	133	TNe+G4	HKY+G
	LSU	863	13	820	46	TNe+I	GTR+I
	rpb2	902	204	565	264	TNe+G4	GTR+G
	SSU	1021	8	1001	46	K2P+I	HKY+I
	b-tub	643	98	463	155	K2P+G4	HKY+G

[x] EF: Translation elongation factor 1-alpha, ITS: Internal transcribed spacer region and intervening 5.8S rDNA gene, LSU: rDNA large subunit, RPB2: RNA polymerase II second largest subunit, SSU: rDNA small subunit, TUB: beta-tubulin; [y] Base substitution models: K2P = Kimura's two-parameter model, TNe = Tamura and Nei model with equal base frequencies, HKY = Hasegawa-Kishino-Yano, and GTR = generalized time-reversible; Non-uniformity among evolutionary sites may be modeled by using: I = proportion of invariable sites, G = Gamma distributed rate variation among sites or the R = FreeRate model (www.iqtree.org/doc/Substitution-Models).
