# Supplementary material for: Kalmusia variispora (Didymosphaeriaceae, Dothideomycetes) Associated with the Grapevine Trunk Disease Complex in Cyprus
Source: Pathogens. 2025 Apr 28;14(5):428. doi: 10.3390/pathogens14050428 (PMC12113838; doi:10.3390/pathogens14050428)
Supplement: Supplementary file 1 [file pathogens-14-00428-s001.zip › Supplementary Table S4.pdf]

Supplementary Table S4. Average daily *in vitro* mycelial growth rate of *Kalmusia variispora* isolates from Cypriot vineyards at 25°C on three different agar-based culture media.

Isolate	Growth rate (mm/day) [x]		
	**PDA**	**MEA**	**OA**
CBS 151327	3.83 ± 0.66	3.51 ± 0.51	4.18 ± 0.61
CBS 151329	3.65 ± 0.40	3.57 ± 0.52	4.06 ± 0.56
CBS 151331	3.98 ± 0.60	3.39 ± 0.50	4.09 ± 0.56
CBS 151334	3.81 ± 0.57	3.13 ± 0.42	4.24 ± 0.64

[x] Fungal cultures were incubated for 14 days, and measurements of colony diameters were recorded daily on potato dextrose agar (PDA), malt extract agar (MEA), and oatmeal agar (OA).
